# Informal carers in severe mental health conditions: Issues raised by the United Kingdom SARS-CoV-2 (COVID-19) Pandemic

**DOI:** 10.1177/0020764020927046

**Published:** 2020-05-10

**Authors:** Juliana Onwumere

**Affiliations:** 1 Department of Psychology, Institute of Psychiatry, Psychology & Neuroscience, King’s College London, London, UK; 2 Bethlem Royal Hospital, South London and Maudsley NHS Foundation Trust, Beckenham, UK

Informal carers represent approximately 10% of the population in the United Kingdom (UK) (Carers UK, n.d.; Office of National Statistics, 2019). These carers, who are typically close family members, play an important and unique role in supporting adults with a disability, care needs related to old age or long-term health conditions, to live in community settings and be part of local communities. This support can be even more crucial in severe mental health conditions (e.g. schizophrenia, bipolar affective disorder) where individuals might experience hallucinations (e.g. hearing the voice of someone speaking to you when no one is there), delusions (e.g. persecutory beliefs about (un)known other trying to do you harm), cognitive disturbance (e.g. difficulties in organising and clearly expressing thoughts), and marked difficulties with social functioning and networks. The course and outcomes of these problems are often significantly improved with carer support (Kuipers et al., 2010).

Carers often help relatives living with severe mental health conditions to engage with treatment programmes, including psychosocial and pharmacological interventions. During different times of the day, carers can be called upon or remain on standby to offer a listening ear and provide encouragement. Indeed, all too often carers will be first in line to provide valued social contact for relatives, who often experience high levels of social exclusion, isolation, loneliness, and stigma (Michalska da Rocha et al., 2018). When compared with the general population, adults with severe mental health conditions have poorer physical health and a reduced life expectancy of approximately 15–20 years. This picture is mostly due to modifiable lifestyle risk factors that are highly prevalent in patient groups such as elevated levels of smoking, sedentariness, poor nutrition, and lower engagement in preventive health behaviours (Hayes et al., 2017). Carers often actively contribute to and support healthier lifestyles in their relatives through different approaches that can include verbal encouragement to make better health choices and directly purchasing, providing, and preparing nutritious meals. They will also facilitate attendance to physical health appointments and act as a buddy to support engagement with physical activities, including going for walks (Onwumere et al., 2018). Finally, as part of their role, carers are also adept at noticing early indicators of deterioration in their relative. They are instrumental in advocating for, and organising, relevant service input and support. Hence, support from carers is linked to significantly reduced rates of psychiatric hospital admissions and admission lengths (Norman et al., 2005).

## Caregiving and UK Coronavirus pandemic: hidden needs

In times of epidemics, pressure on carers is likely to increase as formal health becomes with the spread and control of infection. The global outbreak of novel coronavirus (COVID-19) and its recent arrival in the United Kingdom has led to a significant impact on National Health Service (NHS) capacity and provision, with parallel and unprecedented changes to all sectors of society including employment (e.g. home working), education (e.g. closure of schools/colleges/university, home schooling), leisure (e.g. closure of restaurants, gymnasiums), religious worship (e.g. church closure), social networks (e.g. social (physical) distancing, self-isolation, shielding, lockdown), and individual liberties (e.g. Coronavirus Act, 2020). The Coronavirus Act (2020), for example, is emergency legislation that includes granting the government powers to enforce the detainment for screening, assessment and containment of potentially infectious persons.

It is unclear to what extent such changes to the way individuals are instructed to live their day-to-day lives and implemented at pace from government, as part of an overall public health approach, take account of and impact the experiences and needs of informal caregivers. This is a particularly important area for consideration in severe mental health conditions, where those diagnosed might also fundamentally misunderstand or misinterpret public health information and the recommended guidance on reducing risk of contracting or transmitting COVID-19, and responding in a timely manner to early symptom presentation (e.g. high temperature).

As health care providers, it also remains unclear about how much we have considered the specific COVID-19 related information, support and care needs for carers living in the same household as their relative with a severe mental health condition. Many carers will have been left with the responsibility of sharing public health messages with relatives about the need and importance for social (physical) distancing and of adopting greater hygiene measures (e.g. hand washing) to reduce virus transmission risk. In some cases, carers would have had to lead on implementing shielding^1^ while simultaneously considering any concerns and worries their relative might already have about hidden health threats, risk of harm from others and undertaken in the context of, potentially, elevated levels of suspiciousness and proneness to conspiracy beliefs.

From a clinical and research perspective, and to support messaging strategies for potential future pandemics, it would seem important to know how carers have explained to their relatives what the virus is and its impact, the challenges they have encountered in their communications, and how these have been managed. Moreover, for carers who are not co-resident with their relative and might themselves also be subject to shielding, exploring the intersectionality of physical distancing and mental health caregiving is an area that requires a more in-depth understanding. This is particularly relevant for relationships where the carer and/or their relative are not online and/or familiar with using social media platforms for communication. Exploring the adaptations they might have made to how their relationship works and the implications for their own functioning and quality of the caregiving relationship would also be important.

The UK casualties of the pandemic have been diverse. However, there are some groups that appear to be more affected, specifically those with reported underlying health conditions and those aged over 70 years (i.e. senior citizens). The majority of carers in severe mental health conditions are female, often parents, and likely to be at least middle aged or older. They also report significantly high levels of social isolation compared with the general population and other carer groups (Hayes et al., 2015). While it remains an area often overlooked, carers’ own physical health can be poor and include chronic health conditions such as hypertension (Perlick et al., 2005).

More information is therefore needed on how conversations are facilitated with carers, who are already isolated, about their own health status and concerns they have about the virus and impact on their caregiving role, particularly if they were to become infected. Outside of COVID-19, we know that carers will usually be concerned about who would assume a caregiving role if they were no longer able to provide care and what might become of their relative in the longer term.

In this global pandemic and without an approved vaccine in the short-term, resources and health care professional time will inevitably be focused on prioritised or vulnerable groups. However, it is less clear who could and should assume responsibility or has capacity for thinking about and responding to the specific needs of mental health carers, many of whom will be vulnerable to poor health outcomes. In many health care systems, such carers are typically the last on the list of priorities. There are also sub-groups of carers who are likely to experience even greater levels of needs. For example, carers of adults with comorbid conditions such as substance misuse and/or violence and aggression, younger carers, carers of psychiatric inpatients, and those with more than one caregiving role. Likewise, efforts to understand the cultural and socio-economic framework of COVID-19 and carers’ experiences must not be overlooked. Early indications from UK data speak to the significantly greater proportion of COVID-19 deaths recorded in Black, Asian and minority ethnic (BAME) groups (Intensive Care National Audit and Research Centre, 2020). These are groups also more likely to be diagnosed with severe mental health conditions, particularly within Black African and Caribbean communities, and experience adverse care pathways, poorer care experiences and outcomes (Jongsma et al., 2019; Morgan et al., 2017). Expanding our research and clinical understanding of the intersectionality of ethnicity, social inequalities, and caregiving experiences is recommended.

The important role that carers play and will continue to, beyond the pandemic, means that greater clinical and research efforts must be focused on identifying their unique needs for information, support, and optimal approaches to intervention. The pandemic and its negative effects on individuals, institutions and wider society will be felt for some time. It is therefore essential that as a society we are able to recognise and consider the psychological, physical, and social vulnerability of mental health carers and coordinate and scale up clinical and research efforts to improve their experiences and outcomes from the start. Only this approach can help to prevent even greater morbidity for carers and patients in the future.

## Notes

1. Additional high-level measures to shield those identified on medical grounds as being extremely vulnerable from COVID19 (e.g. not allowed to leave home for 12 weeks).
